# Supplementary material for: Potential plant extinctions with the loss of the Pleistocene mammoth steppe
Source: Nat Commun. 2025 Jan 14;16:645. doi: 10.1038/s41467-024-55542-x (PMC11733255; doi:10.1038/s41467-024-55542-x)
Supplement: Supplementary file 1 — Supplementary Information [file 41467_2024_55542_MOESM1_ESM.pdf]

Potential plant extinctions with the loss of the Pleistocene mammoth steppe

Supplementary Information

Jérémy Courtin[1], Kathleen R. Stoof-Leichsenring[1], Simeon Lisovski[1], Ying Liu[1], Inger Greve Alsos[2], Boris K. Biskaborn[1], Bernhard Diekmann[1], Martin Melles[3], Bernd Wagner[3], Luidmila Pestryakova[4], James Russell[5], Yongsong Huang[5] & Ulrike Herzschuh[1,6,7]*

[1]Polar Terrestrial Environmental Systems, Alfred Wegener Institute Helmholtz Centre for Polar and Marine Research, Potsdam, Germany

[2]The Arctic University Museum of Norway, UiT - The Arctic University of Norway, Tromsø, Norway

[3]Institute of Geology and Mineralogy, University of Cologne, Cologne, Germany

[4]Institute of Natural Sciences, North-Eastern Federal University of Yakutsk, Yakutsk, Russia

[5]Department of Earth, Environmental and Planetary Sciences, Brown University, Providence, USA

[6]Institute of Environmental Science and Geography, University of Potsdam, Potsdam, Germany

[7]Institute of Biology and Biochemistry, University of Potsdam, Potsdam, Germany

***Correspondence**

Ulrike Herzschuh, ulrike.herzschuh@awi.de

Supplementary table 1 - Sequencing devices used for each core.

Supplementary note 1 - Time-slice metrics before resampling.

Supplementary figure 1 - Number of samples per time-slice for each investigated core.

Supplementary table 2 - Metrics of the 2000-year time-slices before resampling.

Supplementary note 2 - Identification of extinct taxa.

Supplementary table 3 - General information about the dbtaxa passing the filters.

Supplementary figure 2 - Density of distribution of occurrences of taxa in GBIF covered (dbtaxa) and not covered (non-dbtaxa) by the SibAla_2023 database.

Supplementary figure 3 - Relative proportion of plant types (colour) per time-slice.

Supplementary table 4 - The species list of 7 ASVs which matches 100% with NCBI, species overlap between the occurrence regions and the northeast Siberia and Alaska region, belonging to the same family.

Supplementary figure 4 - Estimates of species loss.

Supplementary note 3 - Identification of loss of dbtaxa and loss of non-dbtaxa events with other expected rates.

Supplementary figure 5 - Different thresholds used to test the expected plant taxa-loss curve.

Supplementary figure 6 - Different thresholds used to test the expected loss of db-taxa curve.

Supplementary figure 7 - Different thresholds used to test the expected loss of non-dbtaxa curve.

Supplementary note 4 - Choices of best beta-diversity metric and climatic reconstruction estimates to correlate to plant extinction rates.

Supplementary figure 8 - Correlation plot of all parameters tested for correlation.

Supplementary table 5 - Best-fit model when testing all six beta-diversity indices to explain the changes in plant taxa-loss rates.

Supplementary table 6 - Best-fit model when testing all simulated, MAT, and WA-PLS mean and median values per time-slice to explain the changes in plant taxa-loss rates.

Supplementary table 7 - Contributions of the best-fit factors to the observed plant taxa loss in a generalised linear mixed effect model to account for both the simulations and the temporal autocorrelation.

Supplementary note 5 - Characteristics of the lost non-dbtaxa.

Supplementary figure 9 - Distribution of a) number of reads and b) number of samples for the lost non-dbtaxa and the modern non-dbtaxa when using the maximum values of the lost non-dbtaxa as a cut-off for the modern non-dbtaxa.

Supplementary figure 10 - Characterisation of potentially lost plant taxa with the original distribution of the 1000-time resampled data summarised as boxplots.

Supplementary figure 11 - Distribution of a) number of reads and b) number of samples for the lost non-dbtaxa and the modern non-dbtaxa when using the 95% quantile values of the lost non-dbtaxa as a cut-off for the modern non-dbtaxa.

Supplementary table 8 - Comparison of number of reads and number of samples between the lost non-dbtaxa and the modern non-dbtaxa when using the maximum values of the lost non-dbtaxa as a cut-off for the modern non-dbtaxa.

Supplementary table 9 - Comparison of number of reads and number of samples between the lost non-dbtaxa and the modern non-dbtaxa when using the 95% quantile values of the lost non-dbtaxa as a cut-off for the modern non-dbtaxa.

Supplementary table 1
Sequencing devices used for each core.

Core ID	Site - Lake	Project number	Company	Sequencing year	Sequencing device
PG1755	Bilyakh	ALRK-8	Fasteris SA (Switzerland)	2018	Illumina NextSeq 2x150bp Yield - 3GB
PG2133	Bolshoe Toko	HUA-9	Fasteris SA (Switzerland)	2017	Illumina HiSeq 2x125bp Yield - 4.6GB
E5-1A	E5	ALRK-13	Fasteris SA (Switzerland)	2020	Illumina NextSeq 2x150bp Yield – 13.2GB
Co1412	Emanda	APMG-42	Fasteris SA (Switzerland)	2021	Illumina NextSeq 2x150bp Yield – 7.8GB
EN18208	Ilirney	ALRK-10	Fasteris SA (Switzerland)	2020	Illumina NextSeq 2x150bp Yield – 10.2GB
16-KP-01-L02	Ilirney	ALRK-5	Fasteris SA (Switzerland)	2019	Illumina NextSeq 2x150bp Yield – 20.1GB
Co1401	Levinson Lessing	ALRK-11	Fasteris SA (Switzerland)	2020	Illumina NextSeq 2x150bp Yield – 9.3GB
EN18218	Rauchuagytgyn	APMG-30	Fasteris SA (Switzerland)	2021	Illumina NextSeq 2x150bp Yield – 8.2GB

Supplementary note 1

Time-slice metrics before resampling

A 2000-year step was chosen for the time-slices (**supplementary table 2**), as the information carried by 1000-year time-slices was too scarce to carry a robust investigation after resampling. Resampling is performed on the minimum value of 4.319.479 read counts, 7 core counts, and 18 sample counts per time-slice: too much information would have been lost with shorter time-slices (**supplementary figure 1**).

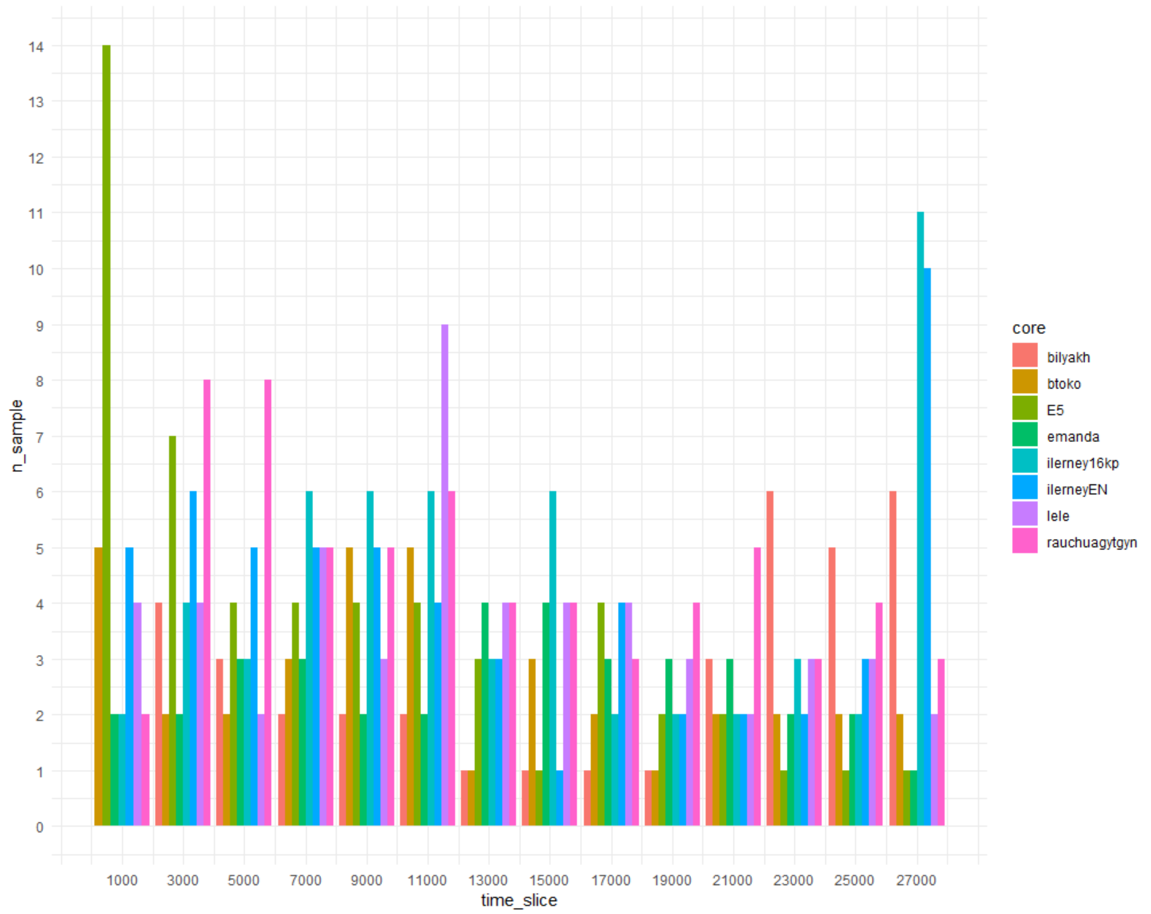

Supplementary figure 1
Number of samples per time-slice for each investigated core.

Supplementary table 2

Metrics of the 2000-year time-slices before resampling (used in the main research article).

Mean age time-slice (cal. yrs BP)	Maximum age (cal. yrs BP)	Minimum age (cal. yrs BP)	Read counts	Core counts	Sample counts
27000	28000	26000	12844725	8	36
25000	26000	24000	5481402	8	22
23000	24000	22000	4855267	8	22
21000	22000	20000	4668076	8	21
19000	20000	18000	4319479	8	18
17000	18000	16000	5758805	8	23
15000	16000	14000	6135805	8	24
13000	14000	12000	6180988	8	23
11000	12000	10000	8037136	8	38
9000	10000	8000	7603592	8	32
7000	8000	6000	8427784	8	33
5000	6000	4000	6664404	8	30
3000	4000	2000	9796097	8	37
1000	2000	0	5848160	7	34

Supplementary note 2

Identification of extinct taxa

2.1 Taxa identification

In this study, we do not try to detect as many non-dbtaxa (i.e. taxa that are not covered by the data base) as possible. Instead, the developed pipeline is stringent and aims to exclude as many potential chimeric, PCR, and sequencing errors as possible by (1) quality filtering of PCR replicates, (2) Use a cut-off for a minimum of 100 reads assigned to ASVs, (3) Use a cut-off for a minimum of 10 sample occurrences of ASVs, (4) Use a co-occurrence and community-based approach to filter out and collapse the candidate ASV signal into a candidate taxon signal. Within communities, we collapse dbASVs with the same taxonomic assignments as dbtaxa, candidate ASVs with the same taxonomic assignments, and candidate ASVs with similar assignment (up to family level) to dbASVs as dbtaxa.

With these steps, we collapse 23,005 ASVs into 359 taxa. Even with this restrictive approach to identify candidate taxa, 60% of the detected taxa are covered by the SibAla_2023 database (dbtaxa) and 40% are not covered by the database (non-dbtaxa). A non-dbtaxon is absent from the SibAla_2023 database and from any sequence database. This can be for three reasons, 1) it is a PCR or sequencing error, 2) it is simply not covered by the sequence database or, 3) it is likely extinct. The first possibility, we have minimised by quality filtering of PCR replicates, applied thresholds of minimum occurrences for ASV detection and co-occurrence, and instigated a community detection pipeline for taxa detection.

Among the 253 taxa from dbASVs with more than 100 reads (**supplementary table 3**), 127 are assigned to species level, from which, 16 species are represented by more than one unique ASV with a total of 37 ASVs. When using only the 100% ASVs to detect communities, for 14 of those species, their corresponding ASVs are part of the same communities (**supplementary data 4**). Such results highlight that different ASVs from similar species are well clustered together with our community-based approach.

Supplementary table 3

General information about the dbtaxa passing the filters.

ASVs and taxa count info	Assigned at 100% to database
All	367
ASVs with more than 100 reads<br><br>*Taxa from ASVs with more than 100 reads*	332<br>*253*
ASVs part of communities with more than 5 ASVs<br>*Taxa from ASVs part of communities with more than 5 ASVs*	289 (87%)<br>*216 (85%)*

2.2 Are rare taxa likely explaining the high proportion of lost non-dbtaxa?

To test whether the non-dbtaxa we observe only originate from the fraction of GBIF plant species not covered by the SibAla_2023 (non-dbtaxa) or if they hold a potentially extinct species signal, we created a synthetic dataset as explained in the main text and we conclude that by sampling according to the GBIF occurrence of taxa, the expected fraction of dbtaxa/non-dbtaxa would not vary through time and would reflect the actual fraction of GBIF db/non-dbtaxa. From the occurrence distribution in GBIF, and the coverage information we have from the SibAla_2023 database, we can see that the distribution of rare to common taxa is similar between the dbtaxa and non-dbtaxa fraction of GBIF (**supplementary figure 2**). A similar distribution of rare to common taxa in the dbtaxa and non-dbtaxa fraction of GBIF would mean that accumulation of random rare taxa loss through several time-slices would not affect drastically the ratio of db/non-db-taxa of GBIF.

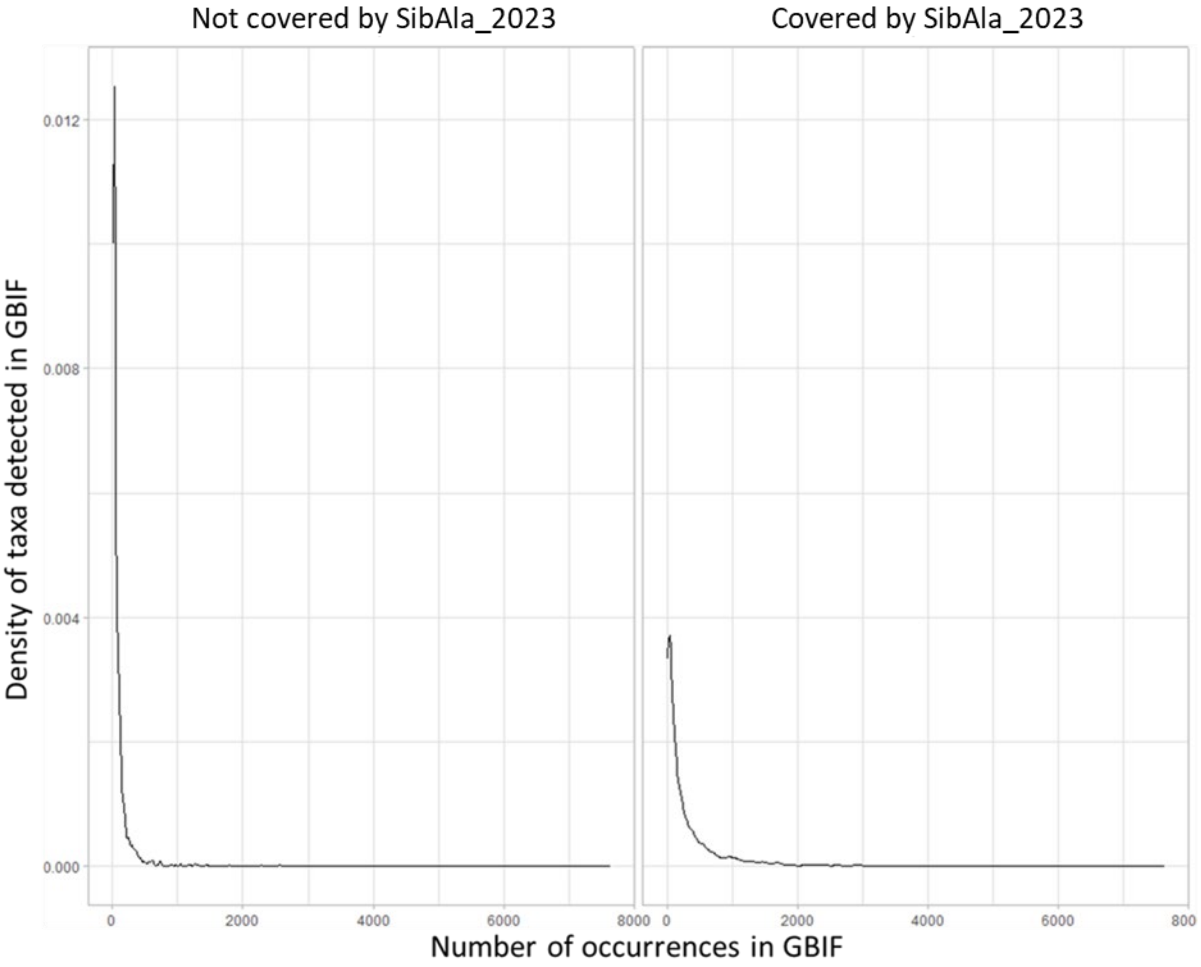

Supplementary figure 2
Density of distribution of occurrences of taxa in GBIF covered (dbtaxa) and not covered (non-dbtaxa) by the SibAla_2023 database.

2.3 Can the non-dbtaxa only be explained by the fraction of modern taxa missing in GBIF?

Another line of evidence to support that non-dbtaxa represent more than the not-covered fraction of GBIF by the sequence database, is the decrease in the proportion of candidate taxa represented relative to the dbtaxa from the oldest time-slice toward the most recent one (**supplementary figure 3**). In the samples from the Pleistocene (~27,000 to ~17,000 cal. yrs BP), 78.3% of the taxa were either forbs or graminoids, with more than 42% of the overall reads assigned. After the LGM, between ~15,000 and ~9,000 cal. yrs BP, there was a shift towards more shrub and tree taxa in the study area. Between ~7,000 and ~1,000 cal. yrs BP, 29.5% of all taxa are assigned to shrubs or trees with more than 91% of the overall reads assigned. For the dbtaxa we see a small decrease in

forbs (73.5% of all dbtaxa before 15,000 cal. yrs BP and 71% after 9,000 cal. yrs BP) and graminoids (11.6% to 8.1%) between the Pleistocene and the Holocene. The non-dbtaxa are generally more impacted with a decrease from 5.5% to 3.1% for graminoids and especially from 61.4% to 42.4% for forbs between before 15,000 cal. yrs BP and after 9,000 cal. yrs BP. This confirms that over time, non-dbtaxa are more often lost in comparison to dbtaxa. Again, if the non-dbtaxa only originated from the not-covered fraction of GBIF by the SibAla_2023 database, we would not expect changes in the proportions between non-dbtaxa and dbtaxa through time as illustrated by the synthetic data.

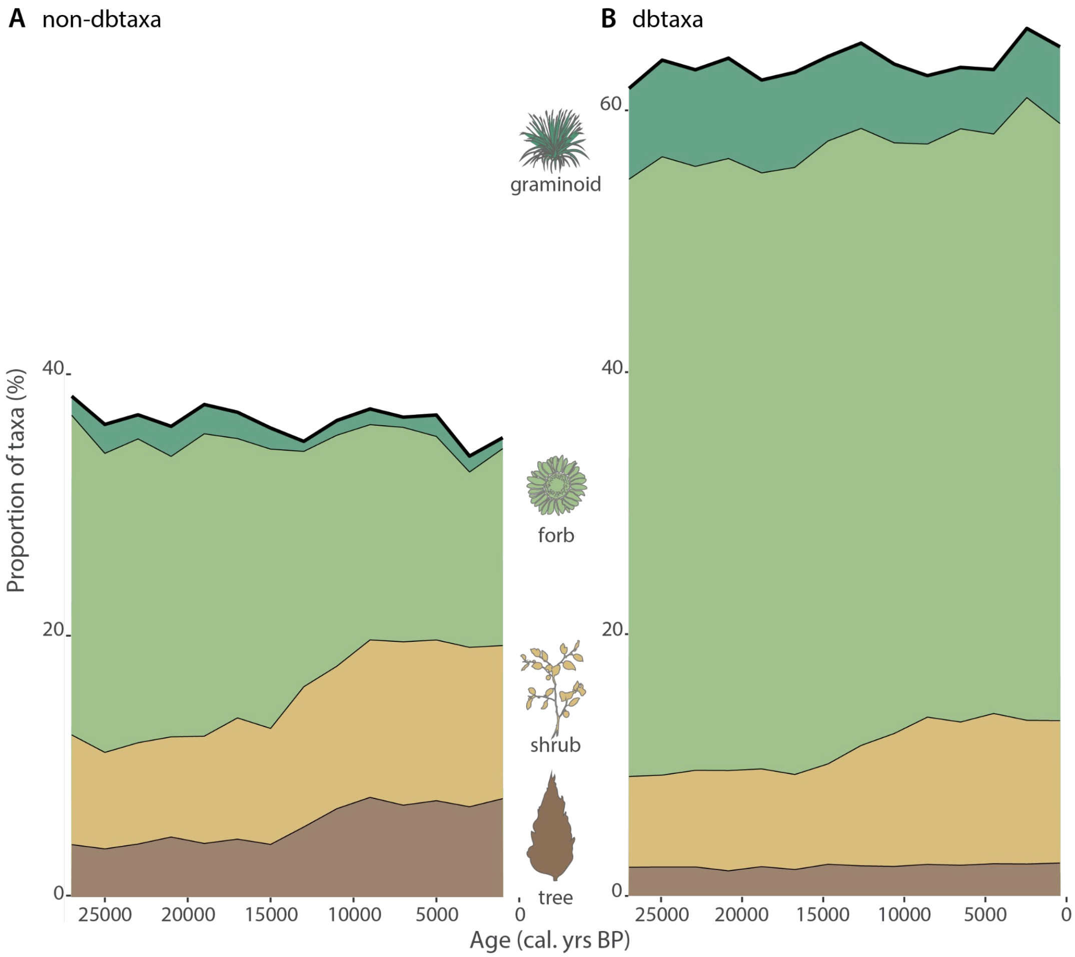

Supplementary figure 3.
Relative proportion of plant types (colour) per time-slice over the last 28,000 years for both the non-*db*taxa (A) and the dbtaxa (B). Graminoids are represented in dark green, forbs in light green, shrubs in yellow, and trees in brown. The bold line highlights the decrease in relative proportion of non-dbtaxa over time.

Supplementary table 4

The species list of 7 non-dbASVs assigned to 6 non-dbtaxa which match 100% with NCBI, species overlap between the occurrence regions and the northeast Siberia and Alaska region, belonging to the same family.

id	query	Length (bp)	match	identity	species present region	species overlap
1	10_cand_Apiaceae_Apioideae	45	*Libanotis buchtormensis*	1	northeast Asia and northwest China	20%(10/50)
1	10_cand_Apiaceae_Apioideae	45	*Libanotis laoshanensis*	1		
1	10_cand_Apiaceae_Apioideae	45	*Libanotis schrenkiana*	1		
1	10_cand_Apiaceae_Apioideae	45	*Tilingia ajanensis*	1		
2	11_cand_Asteraceae_Senecio vulgaris	50	*Arbelaezaster ellsworthii*	1	South America,Southwestern United States	2%(2/100)
2	11_cand_Asteraceae_Senecio vulgaris	50	*Ekmaniopappus sp.*	1		
2	11_cand_Asteraceae_Senecio vulgaris	50	*Filago nevadensis*	1		
2	11_cand_Asteraceae_Senecio vulgaris	50	*Gamochaeta beckii*	1		
2	11_cand_Asteraceae_Senecio vulgaris	50	*Gamochaeta lulioana*	1		
2	11_cand_Asteraceae_Senecio vulgaris	50	*Pseudoclappia arenaria*	1		
2	11_cand_Asteraceae_Senecio vulgaris	50	*Uncultured Streptophyta*	1		
3	11_cand_Boraginaceae_Eritrichium	38	*Eritrichium caucasicum*	1	North Caucasus region	25%(7/28)
4	11_cand_Boraginaceae_Eritrichium	37	*Eritrichium caucasicum*	1	North Caucasus region	25%(7/28)
5	11_cand_Polygonaceae_Rumex	31	*Rumex suffruticosus*	1	Iberian Peninsula	27.6% (8/29)
6	8_cand_Caprifoliaceae_Valeriana officinalis	48	*Adenostyles alliariae*	1	Continental Europe, North America	24% (7/29)
6	8_cand_Caprifoliaceae_Valeriana officinalis	48	*Valeriana apula*	1		
6	8_cand_Caprifoliaceae_Valeriana officinalis	48	*Valeriana dioica*	1		
6	8_cand_Caprifoliaceae_Valeriana officinalis	48	*Valeriana pyrenaica*	1		
6	8_cand_Caprifoliaceae_Valeriana officinalis	48	*Valeriana saliunca*	1		
7	8_cand_Rosaceae_Potentilla	52	*Potentilla ancistrifolia*	1	East Asian Region	4% (4/100)
7	8_cand_Rosaceae_Potentilla	52	*Potentilla dickinsii*	1		

2.4 Do lost non-dbASVs have refugia outsite SibAla region i.e. can they be assigned to EMBL, arctborbryo, PhyloNorway or NCBInt with 100% confidence?

A total of 143 non-dbtaxa are identified from 1028 non-dbASVs. Among them, 16 taxa (11%) and 20 ASVs (2%) match 100% to modern databases (arctborbryo (1, 2, 3); EMBL 143 (4); PhyloNorway (5); **supplementary table 4**). Among the 60 lost non-dbtaxa from 161 non-dbASVs, 6 taxa (10%) and 7 ASVs (4%) matched against the against NCBI after a BLAST query with both 100% similarity and 100% query cover. For those, we identified the occurrence region of matched

species. We compared all species within the same family as the matched species between the occurrence region and the Siberia and Alaska regions to determine the species overlap. As described in the main text, we determined that on average 20% of them have refugia outside of the study area. This means that 20% of 7 taxa (1.4 taxa) are likely not extinct. This suppose that 16.6 (18-1.4) taxa are likely extinct (**supplementary table 4**).

2.5 Can loss of non-dbAVS represent species loss?

To assess whether ASV loss reflects taxa loss and ultimately species loss, we use database data to model the progression of ASV loss. The database may contain cases where one species is represented by multiple sequences, or where a single sequence is linked to multiple species. This mirrors the complex relationship between species and ASVs. As there are 14 time-slices within the *sed*aDNA dataset which we used to estimate potential extinction, to simulate the progress, we set 14 artificial timeslices like the *sed*aDNA dataset (set 14 times ASVs were removed randomly from the database). For every time, the removed number of ASVs are the same as the number of ASVs loss derived from the *sed*aDNA dataset. For example, if there are 100 ASVs loss from the *sed*aDNA dataset in time-slice 14, we also set 100 ASVs loss from the database for the first time. As the corresponding relationships between ASVs and species from the database, which support us examine the number of species losses during each artificial timeslice (one ASV loss does not necessarily equate to the loss of the corresponding species, as there may be other ASVs corresponding to the same species, only all the ASV that corresponding to the species are lost, the species loss be confirmed). We employ the same method as the potential extinction calculation outlined in the main text to determine the proportion of observed species loss to expected species loss. Our analysis reveals a consistent pattern of species loss, showing a slight increase at 17 ka BP and peaking at 9 ka BP and. This result highlights that detection of ASV loss can be interpreted as representing species loss (**supplementary figure 4**).

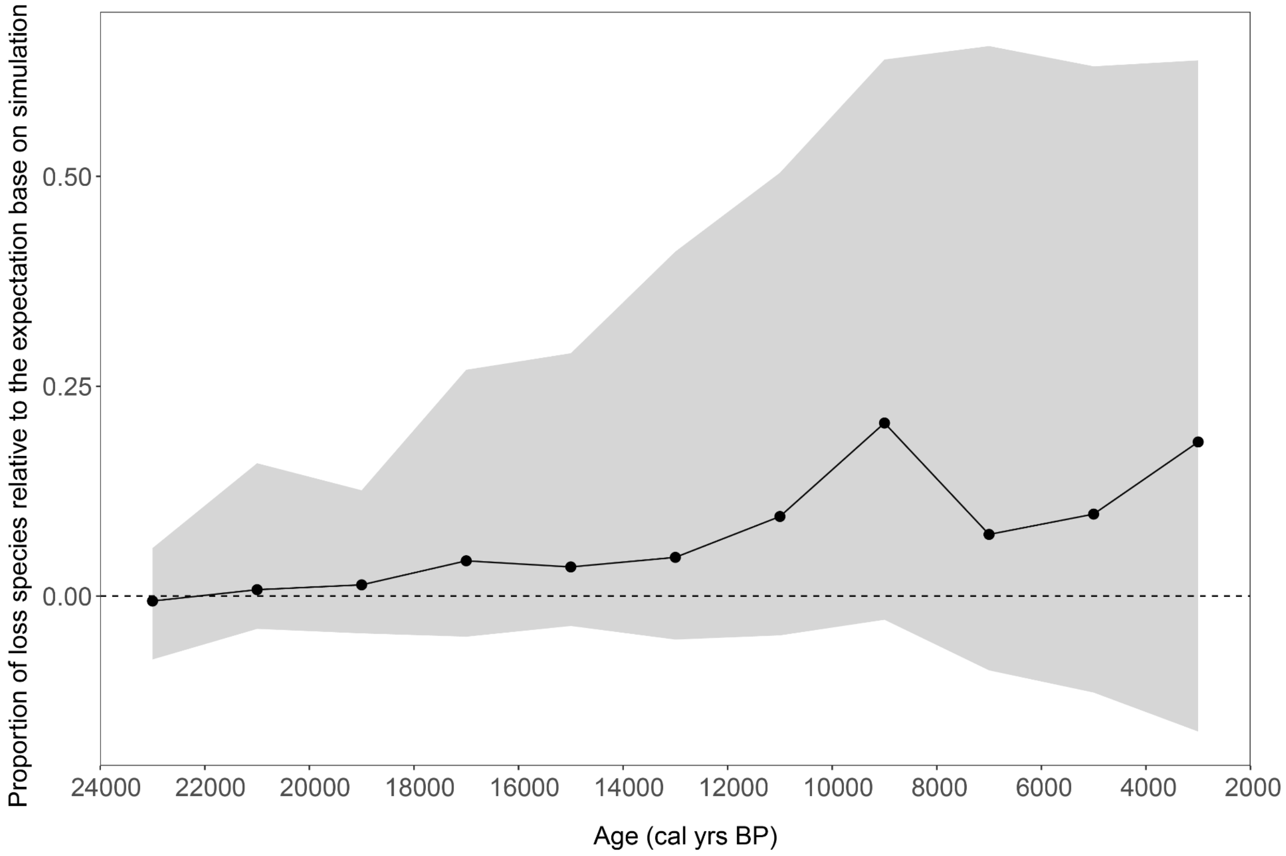

Supplementary figure 4.
Estimates of species loss as a proportion of observed species loss relative to expected species loss. The species loss was inferred from the ASV loss in the artificial timeslice, which has the same number as the non-dbASV loss in the *sed*aDNA dataset.

Supplementary note 3

Identification of loss of dbtaxa and loss of non-dbtaxa events with other expected rates

From 359 Taxa, 242 taxa are present in the modern time slices and are thus identified as not extirpated or extinct. From the remaining 127 taxa, 67 are extirpated, whereas 60 taxa are also absent from the SibAla_2023 database potentially extinct. Based on the dbtaxa only, the extirpation at the seven sites included in the study is 31% while it is of 42% for non-dbtaxa, and thus slightly higher than the extirpation of dbtaxa. As described in **supplementary note 2**, check with global databases and for likelihood of a refugia outside Siberia and Alaska allowed the conclusion that a maximum of 60 non-dbtaxa, and at least 16.6 taxa found in the sediments of the

last 28,000 years, are potentially possibly globally extinct. The potential extinction rate ranges between 5.8 and 1.7 E/MSY over the last 28,000 years in the study area. In comparison, using the 67 dbtaxa lacking from modern time slices, we estimate an extirpation rate of 6.7 extirpation per MSY. If we base the plant loss on each dataset separately, we get a maximum of 11.1 extirpation per MSY for the dbtaxa and 14.6 extirpation and/or extinction per MSY for the non-dbtaxa.

We tested different cut-offs to use as the baseline expected lost dbtaxa and lost non-dbtaxa curve (**supplementary figure 5**). The slices correspond to the expected lost dbtaxa and lost non-dbtaxa curve cut-off. To measure the plant taxa-loss curve, all taxa are accounted (both dbtaxa and non-dbtaxa). The value of the slice is the potential number of time-slices a taxon has to reappear. In the main manuscript, slice nine is used.

For every expected curve measured, the time frame for both the loss of dbtaxa and non-dbtaxa is likely to be between 19,000 cal. yrs BP and 9,000 cal. yrs BP. For all expected curves, the observed loss of dbtaxa is higher than the expected one in time-slices 17,000, 15,000, 13,000, 11,000, and 9,000 cal. yrs BP with a minimum confidence of 90%. The most likely plant taxa loss event is at 13,000 cal. yrs BP with confidence intervals comprising between 95.8% and 99.7%.

We also applied the same approach taking only the dbtaxa for the extirpation curve (**supplementary figure 6**) and only the non-dbtaxa for a potential extinction curve (**supplementary figure 7**). We observe that both the extirpation (**supplementary figure 6**) and the potential extinction (**supplementary figure 7**) time frames with higher observed loss than expected are overlapping and coincide with the time frame identified when using both dbtaxa and candidate taxa (**supplementary figure 5**): especially between 19,000 and 9,000 cal. yrs BP. The peak of extirpation occurs at 13,000 cal. yrs BP while the peaks of potential extinction occur at 17,000–15,000 and 9,000 cal. yrs BP.

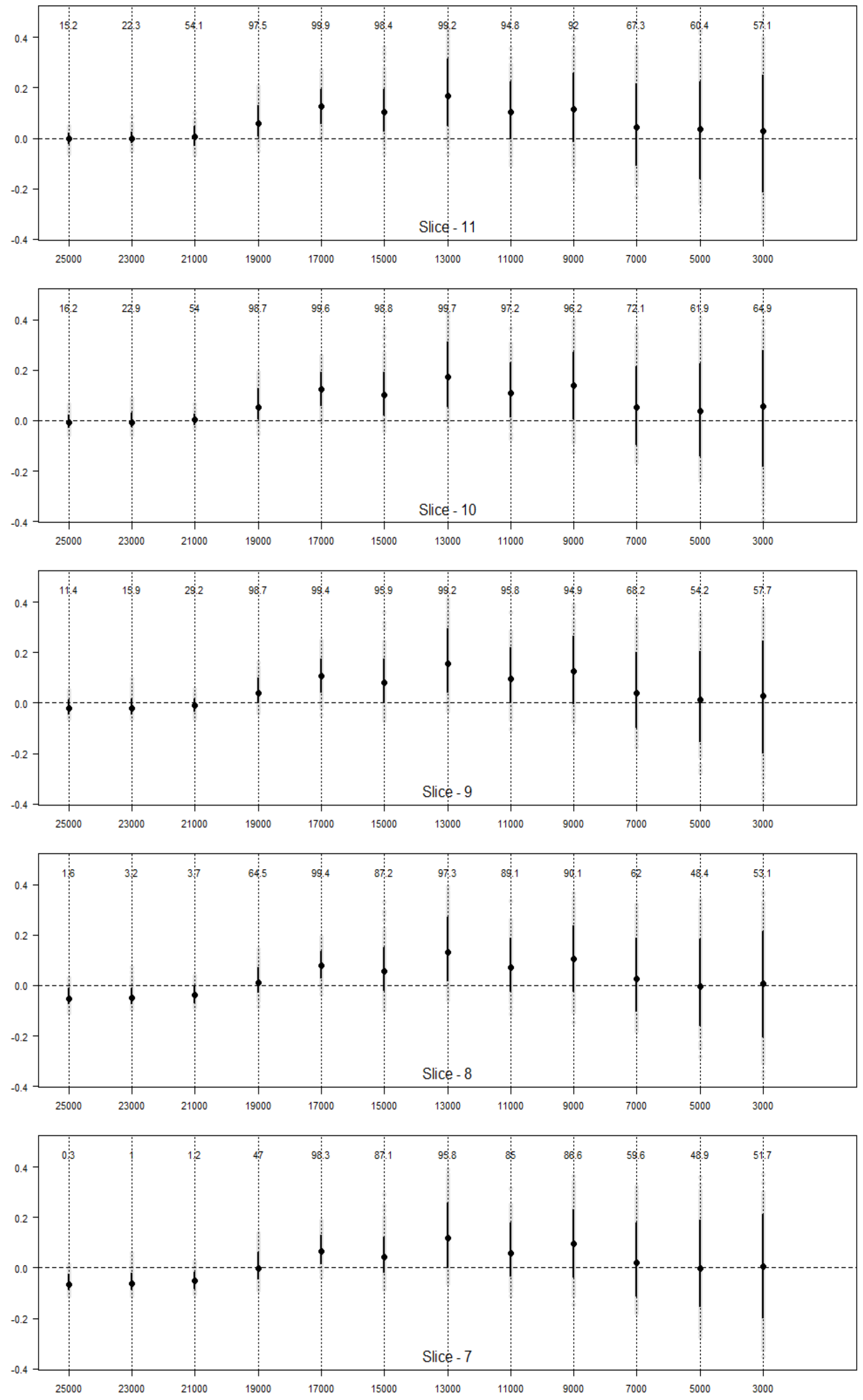

Supplementary figure 5.

Different thresholds used to test the expected plant taxa-loss curve. Based on 100 iterations of the resampling. The black points represent the average values of the distribution (light grey points). The bars delimit the 25% to 75% quantiles of this distribution.

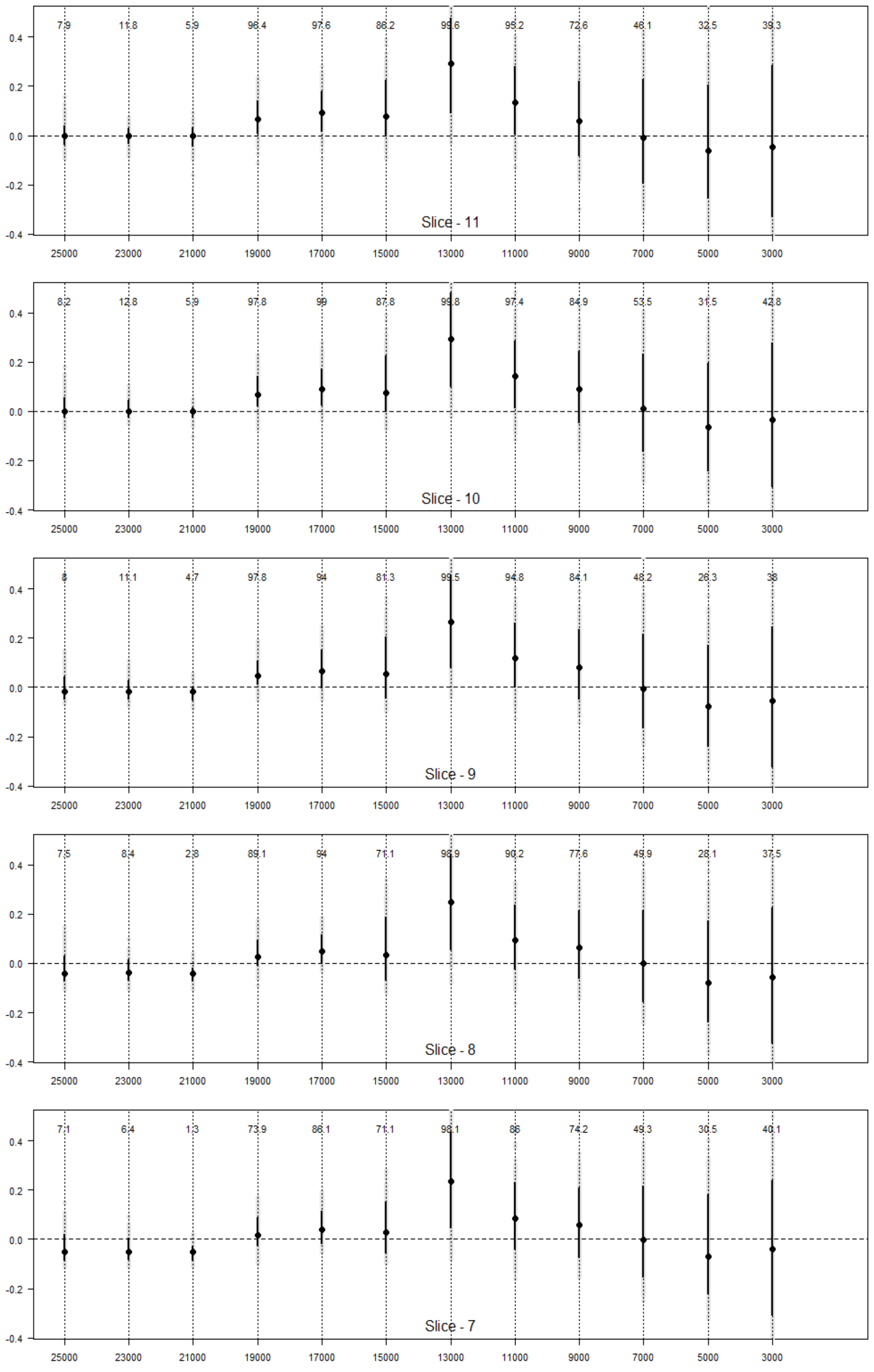

Supplementary figure 6.

Different thresholds used to test the expected loss of db-taxa. Based on 1000 iterations of the resampling. The black points represent the average values of the distribution (light grey points). The bars delimit the 25% to 75% quantiles of this distribution.

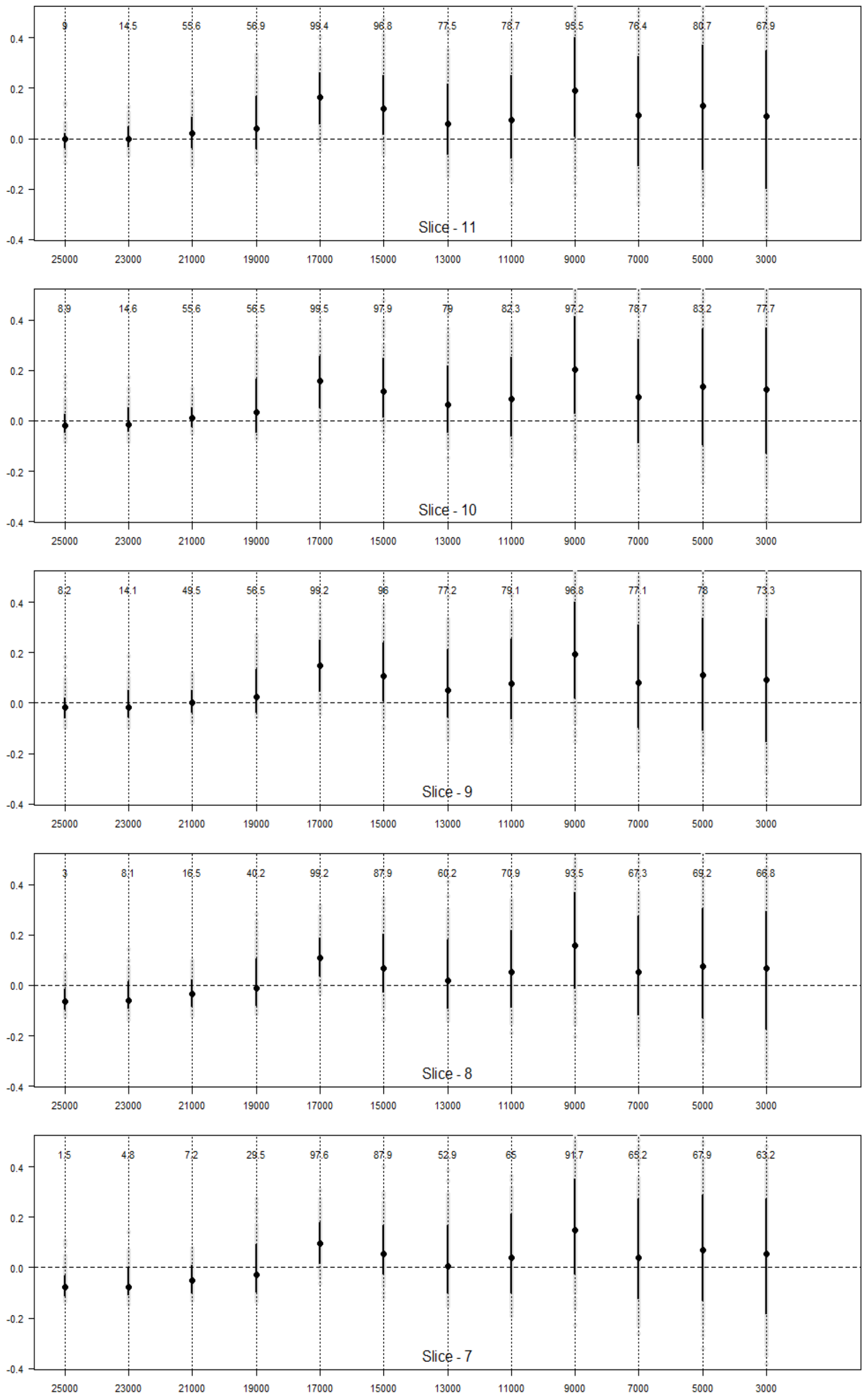

Supplementary figure 7.

Different thresholds used to test the expected loss of non-dbtaxa curve. Based on 1000 iterations of the resampling. The black points represent the average values of the distribution (light grey points). The bars delimit the 25% to 75% quantiles of this distribution.

Supplementary Note 4

Choices of best beta-diversity metric and climatic reconstruction estimates to correlate to plant extinction rates

4.1 Correlation of plant taxa extirpation to potential plant taxa extinction

The difference between the observed loss of dbtaxa and the expected loss of dbtaxa gives us the long-term extirpation rate. In addition, the difference between the observed loss of non-dbtaxa and the expected loss of non-dbtaxa at each time slice gives us the potential plant extinction rate. We measured both rates for each of the 1000 resampling iterations. We observe a slight positive correlation between the plant taxa extirpation rate and the potential plant taxa extinction rate (Spearman, $R = 0.36$, $p < 0.001$; Pearson, $R = 0.21$, $p < 0.001$).

4.2 Correlation of plant taxa-loss rates to beta-diversity metrics

The beta-diversity Jaccard dissimilarity and replacement rate differences between time-slices were measured for each of the 1000 resampling iterations. All were independently correlated with the estimated plant taxa-loss rates. We tested for the beta-diversity factor that explains the best plant taxa-loss rates by building generalised linear models (GLMs) for the dissimilarity and replacement rates with and without positive or negative time lags.

For the loss of dbtaxa rates (extirpation rates), the best-fit model uses only the dissimilarity with a minus one time-slice lag, replacement rates, replacement rates minus one time-slice lag, and replacement rate plus one time-slice lag (**supplementary table 5**). The main explanatory factor is the replacement rate minus one time-slice lag followed by the replacement rates. For the loss of non-dbtaxa rates (potential extinction rates), the best-fit model uses the dissimilarity, the replacement rate minus one time-slice lag, and the dissimilarity minus one time-slice lag (**supplementary table 5**). The dissimilarity minus one time-slice is the main explanatory factor for the potential extinction rate in the model. The replacement rate is a component of the beta-diversity dissimilarity index, and the replacement rate minus one time-slice lag is one of the main explanatory factors for both extirpation and potential extinction rates. We use this last factor as the best explanatory factor in the main manuscript to compare the plant taxa-loss rates to changes in beta-diversity. In the main manuscript, we call vegetation turnover the replacement rate and shifted vegetation turnover is the replacement rate minus one time-slice shift.

All correlations account for autocorrelation (**supplementary figure 8**).

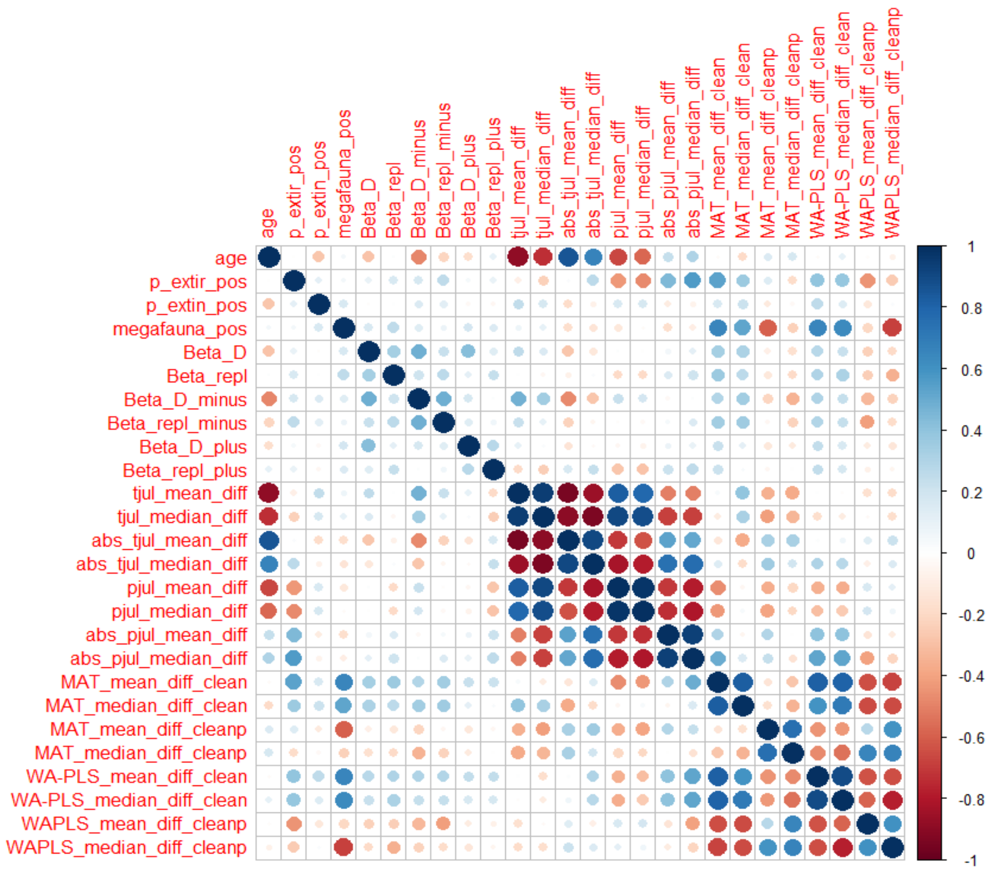

Supplementary figure 8.

Correlation plot of all parameters tested for correlation. Indication about potential autocorrelation corrected. Parameters: age = time-slice; p_extir_pos = positive loss of db-taxa (extirpation rates - negatives are set as 0); p_extir_pos = positive loss of non-dbtaxa (potential extinction rates - negatives are set as 0); megafauna_pos = number of megafauna extinction per time-slice (Stuart, 2015). Beta-diversdity estimates: Beta_D = dissimilarity; Beta_repl = replacement rate; Beta_D_minus = dissimilarity from one time-slice before; Beta_repl_minus = replacement rate from one time-slice before; Beta_D_plus = dissimilarity from one time-slice after; Beta_repl_plus = replacement rate from one time-slice after. Climate estimates, simulated: tjul_mean_diff = mean July temperature anomalies; tjul_median_diff = median July temperature anomalies; abs_tjul_mean_diff = absolute mean July temperature anomalies; abs_tjul_median_diff = absolute median July temperature anomalies; pjul_mean_diff = mean July precipitation anomalies; pjul_median_diff = median July precipitation anomalies; abs_pjul_mean_diff = absolute mean July precipitation anomalies; abs_pjul_median_diff = absolute median July precipitation anomalies. Climate estimates: pollen based: MAT_mean_diff_clean = mean July temperature anomalies using MAT; MAT_median_diff_clean = median July temperature anomalies using MAT; MAT_mean_diff_cleanp = mean July precipitation anomalies using MAT; MAT_median_diff_cleanp = median July precipitation anomalies using MAT; WA-PLS_mean_diff_clean = mean July temperature anomalies using WA-PLS; WA-PLS_median_diff_clean = median July temperature anomalies using WA-PLS; WAPLS_mean_diff_cleanp = mean July precipitation anomalies using WA-PLS; WAPLS_median_diff_cleanp = median July precipitation anomalies using WA-PLS. MAT = modern analogue technique; WA-PLS = weighted-averaging partial least squares.

Supplementary table 5.
Best-fit model when testing all six beta-diversity indices to explain the changes in plant taxa-loss rates.

Factor tested	lost dbtaxa		lost non-dbtaxa	
	Likelihood ratio test (LRT)	p-value	Likelihood ratio test (LRT)	p-value
Replacement rate plus one time-slice shift	6.7	0.01	NA	NA
Dissimilarity	NA	NA	2.79	0.09
Replacement rate	12.3	< 0.001	NA	NA
Replacement rate minus one time-slice shift	46.6	< 0.001	11.4	<0.001
Dissimilarity minus one time-slice shift	9.6	< 0.001	32.9	<0.001

4.3 Correlation of plant taxa-loss rates with climatic condition changes

We tested the simulated temperature and precipitation estimates (recovered from 6) with records from the study area covering the last 25,000 years. We used the mean and median July temperature and precipitation anomalies between our studied time-slices. We also tested pollen-based reconstructed temperature and precipitation records from nine sites. For the pollen-based reconstructed climate changes, both modern analogue technique (MAT) and weighted average-partial least square regression (WA-PLS; 7, 8) approaches were tested for best fit.

Both simulated temperature and precipitation as well as simulated mean and median temperature and precipitation differences between time-slices were tested. Similar data for pollen-based reconstructions (both MAT and WA-PLS) were tested.

All parameters that were independently correlated with the estimated plant taxa-loss rates show a general agreement in the correlation between plant taxa-loss rates and climate-change conditions. To decide on which climate estimate to use in the main Fig. 4 of the manuscript, we tested the climate-change estimate that explains the best plant taxa-loss rates by building GLMs for the different estimates of climate reconstruction.

The one factor that best explains the plant taxa-loss rates is MAT pollen-based reconstructed median temperature (**supplementary table 6**). Because pollen-based reconstructed and simulated climatic data yield similar results, we opted for a pollen-based approach. It is known from experience that these data better fit the expected climate change than the simulated datasets. In

addition, the simulated datasets did not cover the entirety of our 28,000 years' timeframe and were not resilient after 21,000 cal. yrs BP. In addition, precipitation reconstructions are generally less resilient than temperature reconstructions but both temperature and precipitation reconstructions fitted the plant taxa-loss rates consistently. For these reasons, we chose to use the pollen-based median temperature reconstruction based on the MAT approach as an explanatory factor for the plant taxa-loss rates.

Supplementary table 6.

Best-fit model when testing all simulated, MAT, and WA-PLS mean and median values per time-slice to explain the changes in plant taxa-loss rates.

Factor tested	lost dbtaxa		lost non-dbtaxa	
	Likelihood ratio test (LRT)	p-value	Likelihood ratio test (LRT)	p-value
tjul_median_diff	30.9	<0.001	39.4	<0.001
abs_pjul_median_diff	15.3	<0.001	NA	NA
tjul_mean_diff	88.6	<0.001	23	<0.001
WAPLS_mean_diff_cleanp	52.8	<0.001	2.1	0.15
MAT_median_diff_clean	29.8	<0.001	31.4	<0.001
WAPLS_median_diff_cleanp	NA	NA	36.7	<0.001
pjul_median_diff	52.9	<0.001	147.7	<0.001
MAT_mean_diff_clean	NA	NA	92	<0.001

4.4 Correlation of plant taxa-loss rates with megafauna extinction

Using the information reported in references 9 and 10, we estimated the number of megafauna species going extinct during our time-slices. We observe a slight positive correlation between the loss of dbtaxa rate and the megafauna extinction events (Spearman, $R = 0.13$, $p < 0.001$; Pearson, $R = 0.03$, $p < 0.001$). We observe a stronger positive correlation between the loss of non-dbtaxa rate and the megafauna extinction events (Spearman, $R = 0.12$, $p < 0.001$; Pearson, $R = 0.12$, $p < 0.001$).

4.5 Contribution of megafauna extinction, climate change, and beta-diversity change to plant taxa loss

When incorporating all the best-fit factors for climate reconstruction, beta-diversity estimates, and megafauna extinction into one generalised linear mixed effect model to account for both the simulations and the temporal autocorrelation (**supplementary table 7**), the temperature has the highest chi^2 and lowest p-value in the dbtaxa (extirpation) model followed by replacement and megafauna with low chi^2 contributions. However, in the non-dbtaxa (extinction) model, the replacement rate minus one time-slice shift has the highest chi^2, followed by the megafauna extinction with the lowest p-value, and finally the temperature changes.

When testing each of the factors for individual correlation, we find a positive correlation between the estimated beta diversity, calculated as the replacement rate between time-slices minus one time-slice shift and the dbtaxa loss rate (GLM with binomial distribution, z-value10,999 = 12.16; $p < 0.001$). In addition, a positive correlation is found between the pollen-based (MAT) reconstructed July temperature anomalies between time-slices and the dbtaxa loss rates (GLM with binomial distribution, z-value11,999 = 16.51; $p < 0.001$). However, no correlation is observed between the estimated megafauna extinction events, using extinction time frames reported in 9 and 10 and the dbtaxa loss rates (GLM with binomial distribution, z-value11,999 = 1.23; p =0.22).

Supplementary table 7.
Contributions of the best-fit factors to the observed plant taxa loss in a generalised linear mixed effect model to account for both the simulations and the temporal autocorrelation.

Factor tested	lost db-taxa		lost non-dbtaxa	
	Chi^2	$pr(>chi^2)$	Chi^2	$pr(>chi^2)$
Intercept	563.8	<0.001	456.9	<0.001
Replacement rate minus one time-slice shift	55.6	<0.001	30.3	<0.001
Megafauna extinction	13.6	<0.001	16	<0.001
MAT median temperature	109.2	<0.001	6.8	0.009

Supplementary note 5
Characteristics of the lost non-dbtaxa
As lost non-dbtaxa are rarer than the other taxa (modern non-dbtaxa and modern and lost dbtaxa), a selection of modern non-dbtaxa was performed to have a comparable abundance and occurrence to the lost non-dbtaxa. They are based on the maximum number of reads assigned to a lost non-dbtaxon and the maximum number of samples a lost non-dbtaxon can occur in (**supplementary table 8**). The distributions are slightly different as the lost non-dbtaxa are rarer, meaning that they have smaller read counts and number of occurrences on average in the dataset (**supplementary figure 9**). We compared the characteristics of the modern non-dbtaxa to the lost non-dbtaxa in extended results (**supplementary figure 10**).
The results do not change when reducing the number of modern non-dbtaxa (**supplementary figure 8**). For example, when the modern non-dbtaxa are based not on the maximal value of the lost non-dbtaxa but on the 95% quantile of read counts and sample appearance of the lost non-dbtaxa the output is very similar (**supplementary table 9** and **supplementary figure 11**).
Even with a reduced number of taxa in the comparison group when using the 95% quantile, the results are similar regarding the tested parameters. The average community size of the lost non-dbatxa is superior to that of the modern non-dbtaxa (39.85 < 33.77, Wilcox, p-value < 0.001). However, the average SCBD scores of the lost non-dbtaxa is superior to those of the modern candidates (6.43e-5 < 5.32e-5, Wilcox, p-value < 0.001).

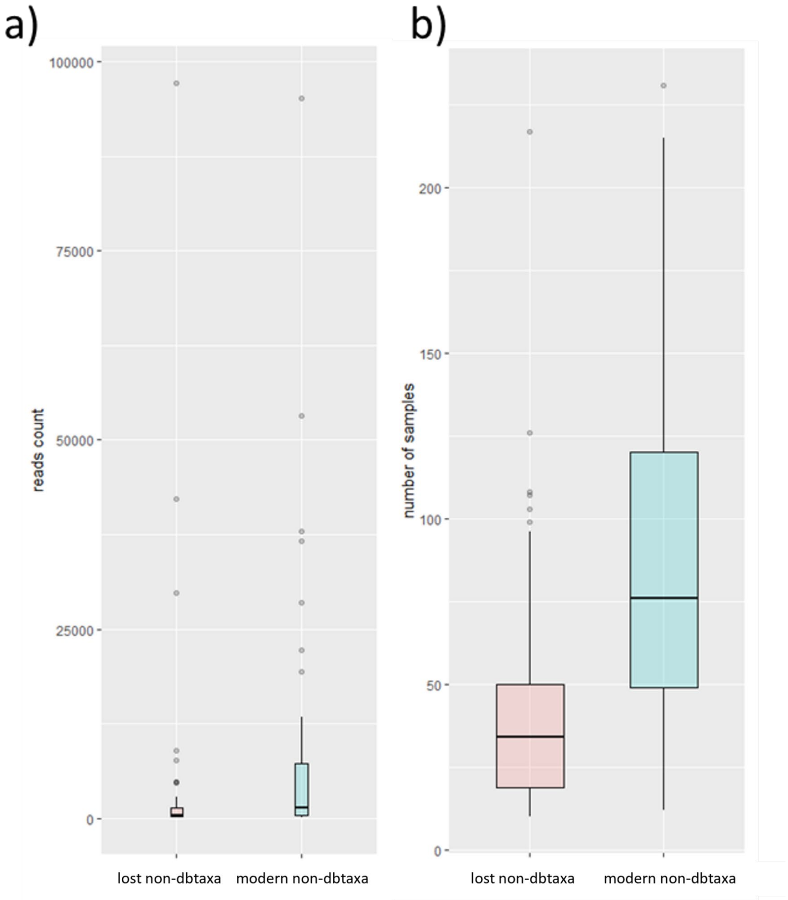

Supplementary figure 9.

Distribution of a) number of reads and b) number of samples for the lost non-dbtaxa and the modern non-dbtaxa when using the maximum values of the lost non-dbtaxa (=60) as a cut-off for the modern non-dbtaxa.

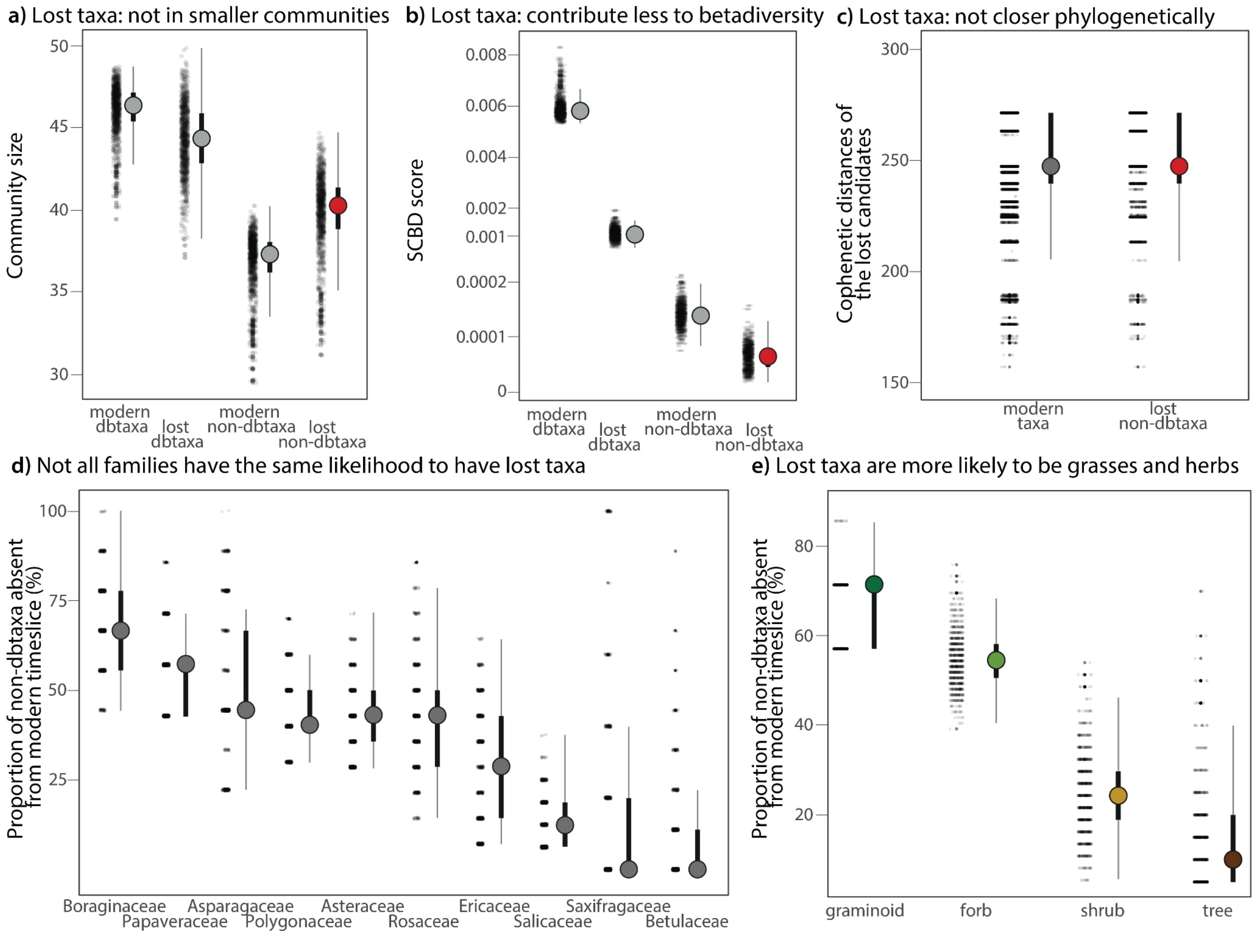

Supplementary figure 10.

Characterisation of potentially lost plant taxa with the original distribution of the 1000-time resampled data (left) summarised as boxplots (right). **a)** Differences in community size between the *db*taxa, the modern non-dbtaxa, and the lost non-dbtaxa; all differences are significant (Wilcoxon test, p-value < 0.001). Lost non-dbtaxa taxa are part of smaller communities compared to *db*taxa. **b)** Differences in species contribution to beta diversity (SCBD) score between the *db*taxa, the modern non-dbtaxa, and the lost non-dbtaxa; all differences are significant (Wilcoxon test, p-value < 0.001). Lost taxa contribute less to beta diversity compared to other taxa. **c)** Cophenetic distances between the lost candidates and between the lost candidates and modern taxa (*db*taxa and modern candidates). The difference is small and not significant (Wilcoxon test, p-value = 0.27). Lost taxa are not phylogenetically closer together than to other taxa. **d)** Proportion of lost candidates relative to the number of candidates for each plant family; all differences are significant (t-test, p-value < 0.001) except for Asteraceae and Rosaceae (t-test, p-value =1). Plant families do not have the same likelihood to have lost taxa. **e)** Proportion of lost candidates relative to the number of candidates for each plant functional type; all differences are significant (t-test, p-value < 0.001). Lost taxa are more likely to be forbs or graminoids than to be shrubs or trees.

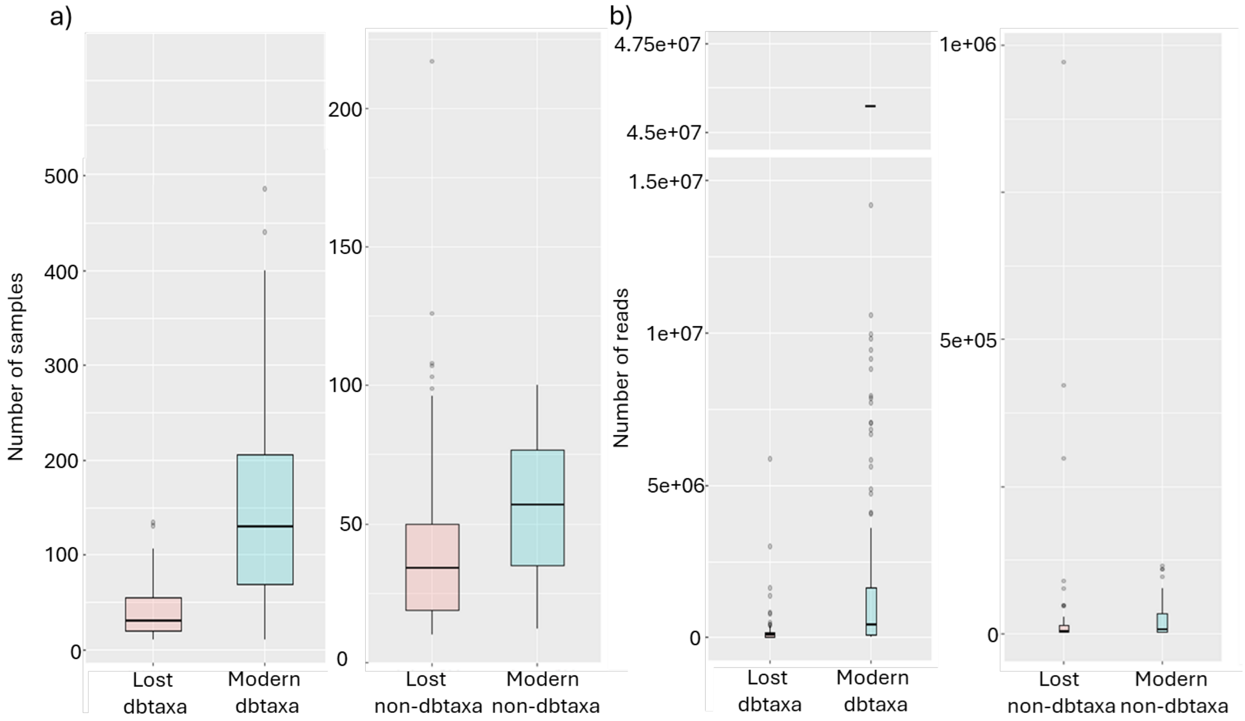

Supplementary figure 11.

Distribution of a) number of reads and b) number of samples for the lost non-dbtaxa and the modern non-dbtaxa when using the 95% quantile values of the lost non-dbtaxa as a cut-off for the modern non-dbtaxa.

Supplementary table 8.

Comparison of number of reads and number of samples between the lost non-dbtaxa and the modern non-dbtaxa when using the maximum values of the lost non-dbtaxa as a cut-off for the modern non-dbtaxa.

Factor tested	lost db-taxa		lost non-dbtaxa	
	Chi^2	pr(>chi^2)	Chi^2	pr(>chi^2)
Intercept	563.8	<0.001	456.9	<0.001
Replacement rate minus one time-slice shift	55.6	<0.001	30.3	<0.001
Megafauna extinction	13.6	<0.001	16	<0.001
MAT median temperature	109.2	<0.001	6.8	0.009

Supplementary table 9.

Comparison of number of reads and number of samples between the lost non-dbtaxa and the modern non-dbtaxa when using the 95% quantile values of the lost non-dbtaxa as a cut-off for the modern non-dbtaxa.

95% quantile	Lost non-dbtaxa	Similar Modern non-dbtaxa	Lost dbtaxa	Modern dbtaxa
Number of taxa	60	46	67	149
Max reads count	97194	9720	586938	46362941
Mean reads count	3842.83	1761.59	29384	766535
Max number of samples	217	106	134	486
Mean number of samples	42.77	56.37	41	149

References

1. E. Willerslev, J. Davison, M. Moora, M. Zobel, E. Coissac, M. E. Edwards, E. D. Lorenzen, M. Vestergård, G. Gussarova, J. Haile, Fifty thousand years of Arctic vegetation and megafaunal diet. *Nature,* 506, 47-51 (2014). https://doi.org/10.1038/nature12921

2. E. M. Soininen, G. Gauthier, F. Bilodeau, D. Berteaux, L. Gielly, P. Taberlet, G. Gussarova, E. Bellemain, K. Hassel, H. K. Stenøien, Highly overlapping winter diet in two sympatric lemming species revealed by DNA metabarcoding. *PLoS One,* 10, e0115335 (2015). https://doi.org/10.1371/journal.pone.0115335

3. J. Sønstebø, L. Gielly, A. Brysting, R. Elven, M. Edwards, J. Haile, E. Willerslev, E. Coissac, D. Rioux, J. Sannier, Using next-generation sequencing for molecular reconstruction of past Arctic vegetation and climate. *Molecular Ecology Resources,* 10, 1009-1018 (2010). https://doi.org/10.1111/j.1755-0998.2010.02855.x

4. C. Kanz, P. Aldebert, N. Althorpe, W. Baker, A. Baldwin, K. Bates, P. Browne, A. van den Broek, M. Castro, G. Cochrane, The EMBL nucleotide sequence database. *Nucleic Acids Research* 33, D29-D33 (2005). https://doi.org/10.1093/nar/gki098

5. I. G. Alsos, D. P. Rijal, D. Ehrich, D. N. Karger, N. G. Yoccoz, P. D. Heintzman, A. G. Brown, Y. Lammers, L. Pellissier, T. Alm, Postglacial species arrival and diversity buildup of northern ecosystems took millennia. *Science Advances,* 8, eabo7434 (2022). https://doi.org/10.1126/sciadv.abo7434

6. A. Dallmeyer, T. Kleinen, M. Claussen, N. Weitzel, X. Cao, U. Herzschuh, The deglacial forest conundrum. *Nature Communications,* 13, 6035 (2022). https://doi.org/10.1038/s41467-022-33646-6

7. U. Herzschuh, C. Li, T. Böhmer, A. K. Postl, B. Heim, A. A. Andreev, X. Cao, M. Wieczorek, J. Ni, LegacyPollen 1.0: a taxonomically harmonized global late Quaternary pollen dataset of 2831 records with standardized chronologies. *Earth System Science Data,* 14, 3213-3227 (2022). https://doi.org/10.5194/essd-14-3213-2022

8. U. Herzschuh, T. Böhmer, C. Li, M. Chevalier, A. Dallmeyer, X. Cao, N. H. Bigelow, L. Nazarova, E. Y. Novenko, J. Park, LegacyClimate 1.0: A dataset of pollen-based climate reconstructions from 2594 Northern Hemisphere sites covering the late Quaternary. *Earth System Science Data Discussions*, 1-29 (2022). https://doi.org/10.5194/essd-2022-38

9. A. J. Stuart, Late Quaternary megafaunal extinctions on the continents: a short review. *Geological Journal*, 50(3), 338-363 (2015). https://doi.org/10.1002/gj.2633

10. G. D. Zazula, E. Hall, P. G. Hare, C. Thomas, R. Mathewes, C. La Farge, A. L. Martel, P. D. Heintzman, B. Shapiro, A middle Holocene steppe bison and paleoenvironments from the Versleuce Meadows, Whitehorse, Yukon, Canada. *Canadian Journal of Earth Sciences*, 54(11), 1138-1152 (2017). https://doi.org/10.1139/cjes-2017-0100
