# Supplementary material for: Potential plant extinctions with the loss of the Pleistocene mammoth steppe
Source: Nat Commun. 2025 Jan 14;16:645. doi: 10.1038/s41467-024-55542-x (PMC11733255; doi:10.1038/s41467-024-55542-x)
Supplement: Supplementary file 3 — Description of Additional Supplementary Files [file 41467_2024_55542_MOESM3_ESM.pdf]

Potential plant extinctions with the loss of the Pleistocene mammoth steppe

Description of Additional Supplementary Files

Jérémy Courtin[1], Kathleen R. Stoof-Leichsenring[1], Simeon Lisovski[1], Ying Liu[1], Inger Greve Alsos[2], Boris K. Biskaborn[1], Bernhard Diekmann[1], Martin Melles[3], Bernd Wagner[3], Luidmila Pestryakova[4], James Russell[5], Yongsong Huang[5] & Ulrike Herzschuh[1,6,7]*

[1]Polar Terrestrial Environmental Systems, Alfred Wegener Institute Helmholtz Centre for Polar and Marine Research, Potsdam, Germany

[2]The Arctic University Museum of Norway, UiT - The Arctic University of Norway, Tromsø, Norway

[3]Institute of Geology and Mineralogy, University of Cologne, Cologne, Germany

[4]Institute of Natural Sciences, North-Eastern Federal University of Yakutsk, Yakutsk, Russia

[5]Department of Earth, Environmental and Planetary Sciences, Brown University, Providence, USA

[6]Institute of Environmental Science and Geography, University of Potsdam, Potsdam, Germany

[7]Institute of Biology and Biochemistry, University of Potsdam, Potsdam, Germany

***Correspondence**

Ulrike Herzschuh, ulrike.herzschuh@awi.de

Supplementary data 1: Data table with the DNA sequence and assignment of the 5129 ASVs (dbASVs and non-dbASVs) with a minimum of 100 reads used in this study and part of communities with more than 5 ASVs.

Supplementary data 2: Data table showing the different compositions of the detected communities.

Supplementary data 3: Data table showing the different taxa detected and their median representation in each timeslice based on the 1000 times resampling step.

Supplementary data 4: Data table of the different taxa represented by more than one ASVs for each community detected.

Supplementary data 5: Input data needed to perform the different analyses and run the R scripts deposited in https://doi.org/10.5281/zenodo.14033305 and https://doi.org/10.5281/zenodo.14033298.
