# Supplementary material for: Potential plant extinctions with the loss of the Pleistocene mammoth steppe
Source: Nat Commun. 2025 Jan 14;16:645. doi: 10.1038/s41467-024-55542-x (PMC11733255; doi:10.1038/s41467-024-55542-x)
Supplement: Supplementary file 9 — Reporting Summary [file 41467_2024_55542_MOESM9_ESM.pdf]

nature portfolio

Corresponding author(s): Ulrike Herzschuh

Last updated by author(s): 05/11/2024

Reporting Summary

Nature Portfolio wishes to improve the reproducibility of the work that we publish. This form provides structure for consistency and transparency in reporting. For further information on Nature Portfolio policies, see our Editorial Policies and the Editorial Policy Checklist.

Statistics

For all statistical analyses, confirm that the following items are present in the figure legend, table legend, main text, or Methods section.

n/a	Confirmed	
☐	☒	The exact sample size (*n*) for each experimental group/condition, given as a discrete number and unit of measurement
☒	☐	A statement on whether measurements were taken from distinct samples or whether the same sample was measured repeatedly
☐	☒	The statistical test(s) used AND whether they are one- or two-sided<br>*Only common tests should be described solely by name; describe more complex techniques in the Methods section.*
☐	☒	A description of all covariates tested
☐	☒	A description of any assumptions or corrections, such as tests of normality and adjustment for multiple comparisons
☐	☒	A full description of the statistical parameters including central tendency (e.g. means) or other basic estimates (e.g. regression coefficient) AND variation (e.g. standard deviation) or associated estimates of uncertainty (e.g. confidence intervals)
☐	☒	For null hypothesis testing, the test statistic (e.g. *F*, *t*, *r*) with confidence intervals, effect sizes, degrees of freedom and *P* value noted<br>*Give P values as exact values whenever suitable.*
☒	☐	For Bayesian analysis, information on the choice of priors and Markov chain Monte Carlo settings
☒	☐	For hierarchical and complex designs, identification of the appropriate level for tests and full reporting of outcomes
☒	☐	Estimates of effect sizes (e.g. Cohen's *d*, Pearson's *r*), indicating how they were calculated

Our web collection on statistics for biologists contains articles on many of the points above.

Software and code

Policy information about availability of computer code

Data collection	All information on data collection are described in the main text or supplementary information.<br><br>For all the 8 sediment cores used:<br>- DNA datasets will be deposited in the European Nucleotide Archive (ENA) under the INSDC accession number: PRJEB76237<br>- Age models used are cited in Table 1:<br>Bilyakh PG1755: https://doi.org/10.1016/j.quascirev.2010.04.024<br>Bolshoe Toko PG2133: https://doi.org/10.3389/fevo.2021.625096<br>E5 E5-1A: https://doi.org/10.1016/j.quascirev.2018.12.003<br>Emanda C01412: https://doi.org/10.1111/bor.12476<br>Ilirney EN18208: https://doi.org/10.1016/j.quascirev.2020.106607<br>Ilirney 16-KP-01-L02: https://doi.org/10.1111/bor.12521<br>Levinson Lessing Co1401: https://doi.org/10.1002/jqs.3384<br>Rauchuagytgyn EN18218: https://doi.org/10.5194/bg-18-4791-2021<br><br>The SibAla_2023 database was built using GBIF information on plant distribution: https://www.gbif.org<br><br>The temperature information used for correlation in figure 3 is a pollen-based median temperature reconstruction based on the MAT approach (https://doi.org/10.5194/essd-14-3213-2022): We also tested a pollen based reconstruction based on WA-PLS (https://doi.org/10.5194/essd-2022-38) and a simulated temperature and precipitation estimates (https://doi.org/10.1038/s41467-022-33646-6), described in supplementary information.
Data analysis	The data analysis used open access softwares.<br>All scripts are deposited on Github under two project. One for the SIbAla_2023 database building process: https://doi.org/10.5281/zenodo.14033298 and the other one for the data analysis under: https://doi.org/10.5281/zenodo.14033305. All required data to perfom the analyses are in the main manuscript or provided in supplementary data 5.<br>DNA sequences were processed and classified with OBITools v3.<br><br>The downstream data handling, statistical analysis, network analysis and plotting was performed in R v4.0.3:<br>DNA replicates were quality checked via NMDS analyses: R package vegan v2.6-2<br>Correlation of occurrence analysis: R package psych v2.2.3<br>Network construction and communities detection: R package igraph v1.3.0<br>Resampling was perfomed with a custom script avaliable on GitHub<br>GLM, spearman, pearson and wilcoxon tests: R v4.0.3.<br>Phylogeny of taxa: R package GBOTB-extended.TPL v0.1.0<br>Cophenetic distances measurments: R package ape v5.6-2

For manuscripts utilizing custom algorithms or software that are central to the research but not yet described in published literature, software must be made available to editors and reviewers. We strongly encourage code deposition in a community repository (e.g. GitHub). See the Nature Portfolio guidelines for submitting code & software for further information.

Data

Policy information about availability of data

All manuscripts must include a data availability statement. This statement should provide the following information, where applicable:

- Accession codes, unique identifiers, or web links for publicly available datasets
- A description of any restrictions on data availability
- For clinical datasets or third party data, please ensure that the statement adheres to our policy

All data needed to evaluate the conclusions in the paper are present in the paper and/or the supplementary information. Final sedaDNA data used are given as supplementary data 3. All newly generated raw sedaDNA sequence data are deposited in the European Nucleotide Archive (ENA) (www.ebi.ac.uk/ena/browser/home) under the INSDC accession number: PRJEB76237. Input files needed to run the different scripts are given in supplementary data 5.

Research involving human participants, their data, or biological material

Policy information about studies with human participants or human data. See also policy information about sex, gender (identity/presentation), and sexual orientation and race, ethnicity and racism.

Reporting on sex and gender	NA
Reporting on race, ethnicity, or other socially relevant groupings	NA
Population characteristics	NA
Recruitment	NA

Ethics oversight	NA

Note that full information on the approval of the study protocol must also be provided in the manuscript.

Field-specific reporting

Please select the one below that is the best fit for your research. If you are not sure, read the appropriate sections before making your selection.

☐ Life sciences ☐ Behavioural & social sciences ☒ Ecological, evolutionary & environmental sciences

For a reference copy of the document with all sections, see nature.com/documents/nr-reporting-summary-flat.pdf

Ecological, evolutionary & environmental sciences study design

All studies must disclose on these points even when the disclosure is negative.

Study description	This study underscores the potential of sedaDNA to investigate historical extinction events. We established a new approach using metabarcoding sequencing of sedimentary ancient DNA from lakes across Siberia and Alaska covering the last 28,000 years to provide first evidence of potential plant extinction at the transition between the Pleistocene and the Holocene. First, we curated a new DNA database (SibAla_2023). Then, we developed a new approach to detect potentially extinct taxa that allow mismatches in DNA taxonomic assignments and groups ASVs and taxa based on co-occurrence patterns and statistically detected communities. Finally, to comprehensively analyse data in 504 samples from 8 lake sediment cores with known age models that we re-sampled in 14 time-slices of 2000 years. After those three major methodological steps, we could identify and quantify potential plant extinction events at the transition between the Pleistocene and the Holocene being above background extinction rates but below modern estimates. We could also correlate potential plant extinction to known events happening during this transition such as, climate change, megafauna extinction or change in plant composition. Finally, our data allows to characterise the plant taxa the most likely to get potentially extinct. The initial estimates provided here serve to pave the way for a deeper understanding of the interactions between rapid ecosystem loss, plant compositional shifts, and cascading extinctions across different taxonomic kingdoms, in the context of climate change and will improve as the paleoecology community continues to expand DNA reference libraries and ancient sedaDNA data.
Research sample	For the final analysis, we used 504 samples from 8 lake sediments cores (described in Table 1). For every core, DNA isolations were performed, each with nine samples and one control (blank). The extraction blank and one no template control (NTC) was included in each PCR to identify possible contamination during extraction and PCR set-up. For each extraction sample, three PCR replicates with differently tagged primers were performed. Except for the Bolshoe Toko core, replicates were amplified with the same tag combination. For each core, all extraction blanks and NTCs were included in the sequencing run, even though they were negative in the PCRs.
Sampling strategy	A re-sampling step was performed to compare the 504 samples from 8 lake sediment cores from Siberia and Alaska (area covered by the now lost mammoth steppe biota) covering at least the last 28,000 years (transition between the Pleistocene and the Holocene). In the end, we worked with 14 samples (covering the 2,000 years each). We set the 2,000 years span for our time step to insure that each time-slice covers most cores (minimum of 7), has enough samples (minimum of 18) and reads (minimum of 4,319,479) to allow a rigorous resampling.
Data collection	The datasets used are open access, cited in the text, and were retrieved from PANGAEA or present in the supplementary information of the corresponding paper. Further newly generated and therefore, not yet available data is available in supplementary data 5, the European Nucleotide Archive (ENA) and GitHub.<br><br>For all the 8 sediment cores used:<br>- DNA datasets will be deposited in ENA<br>- Age models used are cited in Table 1:<br>Bilyakh PG1755: https://doi.org/10.1016/j.quascirev.2010.04.024<br>Bolshoe Toko PG2133: https://doi.org/10.3389/fevo.2021.625096<br>E5 E5-1A: https://doi.org/10.1016/j.quascirev.2018.12.003<br>Emanda C01412: https://doi.org/10.1111/bor.12476<br>Ilirney EN18208: https://doi.org/10.1016/j.quascirev.2020.106607<br>Ilirney 16-KP-01-L02: https://doi.org/10.1111/bor.12521<br>Levinson Lessing Co1401: https://doi.org/10.1002/jqs.3384<br>Rauchuagytgyn EN18218: https://doi.org/10.5194/bg-18-4791-2021<br><br>The SibAla_2023 database was built using GBIF information on plant distribution: https://www.gbif.org<br><br>The temperature information used for correlation in figure 3 is a pollen-based median temperature reconstruction based on the MAT approach (https://doi.org/10.5194/essd-14-3213-2022): We also tested a pollen based reconstruction based on WA-PLS (https://doi.org/10.5194/essd-2022-38) and a simulated temperature and precipitation estimates (https://doi.org/10.1038/s41467-022-33646-6), described in supplementary information.<br><br>Information on megafauna extinction at the late Pleistocene - Holocene transition in our study area was retrieved from https://doi.org/10.1002/gj.2633 and https://doi.org/10.1139/cjes-2017-0100.

Timing and spatial scale	The samples used in this study cover the last 28,000 years. We worked with 14 timeslices of 2000 years. Detail information on the number of samples per time-slice for each investigated core can be find in supplementary figure 1.
Data exclusions	All data handling and exclusion is detailed in the manuscript and supplementary materials.<br><br>For each samples, the three replicates were quality checked. If the total read count of the affected replicate was below 100 counts and/or the sample was composed of fewer (less than three) and different plant sequence types as compared to the remaining replicates of the sample, replicates were excluded from the dataset.<br><br>To investigate potential extinction, we used ASVs information with mismatches to DNA database. We used all ASVs assigned to a taxon to SibAla_2023 with at least 90% match. The other ASVs were excluded. Further, we used different thresholds to insure the quality of the data presented and avoid sequencing or PCR errors of ASVs with mismatches to the SibAla_2023 database. Only ASVs appearing in at least 10 samples and with a minimum of 100 reads were kept. Only ASVs part of detected plant communites (containing at least 5 ASVs) were kept. From the original 23,005 ASVs, 5,129 passed all filters and were used to present the final results.
Reproducibility	Only a part of the prepared sequencing libraries were sequenced and the remainders are frozen at the Alfred Wegener Institute, Potsdam. The data analysis is fully reproducible with the data used deposited and the scripts are deposited in GitHub available to the public under: https://doi.org/10.5281/zenodo.14033298 and https://doi.org/10.5281/zenodo.14033305.
Randomization	The final data presented is based on the median value after a resampling performed 1000 times.
Blinding	*Describe the extent of blinding used during data acquisition and analysis. If blinding was not possible, describe why OR explain why blinding was not relevant to your study.*

Did the study involve field work? ☒ Yes ☐ No

Field work, collection and transport

Field conditions	*Describe the study conditions for field work, providing relevant parameters (e.g. temperature, rainfall).*
Location	*State the location of the sampling or experiment, providing relevant parameters (e.g. latitude and longitude, elevation, water depth).*
Access & import/export	*Describe the efforts you have made to access habitats and to collect and import/export your samples in a responsible manner and in compliance with local, national and international laws, noting any permits that were obtained (give the name of the issuing authority, the date of issue, and any identifying information).*
Disturbance	*Describe any disturbance caused by the study and how it was minimized.*

Reporting for specific materials, systems and methods

We require information from authors about some types of materials, experimental systems and methods used in many studies. Here, indicate whether each material, system or method listed is relevant to your study. If you are not sure if a list item applies to your research, read the appropriate section before selecting a response.

Materials & experimental systems

n/a	Involved in the study	
☒	☐	Antibodies
☒	☐	Eukaryotic cell lines
☒	☐	Palaeontology and archaeology
☒	☐	Animals and other organisms
☒	☐	Clinical data
☒	☐	Dual use research of concern
☒	☐	Plants

Methods

n/a	Involved in the study	
☒	☐	ChIP-seq
☒	☐	Flow cytometry
☒	☐	MRI-based neuroimaging

Plants

Seed stocks	*Report on the source of all seed stocks or other plant material used. If applicable, state the seed stock centre and catalogue number. If plant specimens were collected from the field, describe the collection location, date and sampling procedures.*
Novel plant genotypes	*Describe the methods by which all novel plant genotypes were produced. This includes those generated by transgenic approaches, gene editing, chemical/radiation-based mutagenesis and hybridization. For transgenic lines, describe the transformation method, the number of independent lines analyzed and the generation upon which experiments were performed. For gene-edited lines, describe the editor used, the endogenous sequence targeted for editing, the targeting guide RNA sequence (if applicable) and how the editor was applied.*
Authentication	*Describe any authentication procedures for each seed stock used or novel genotype generated. Describe any experiments used to assess the effect of a mutation and, where applicable, how potential secondary effects (e.g. second site T-DNA insertions, mosiacism, off-target gene editing) were examined.*
